# Reliability and Validity of Quantifying Absolute Muscle Hardness Using Ultrasound Elastography

**DOI:** 10.1371/journal.pone.0045764

**Published:** 2012-09-21

**Authors:** Kentaro Chino, Ryota Akagi, Michiko Dohi, Senshi Fukashiro, Hideyuki Takahashi

**Affiliations:** 1 Department of Life Sciences (Sports Sciences), Graduate School of Arts and Sciences, the University of Tokyo, Meguro-ku, Tokyo, Japan; 2 Department of Sports Sciences, Japan Institute of Sports Sciences, Kita-ku, Tokyo, Japan; 3 Department of Sports Medicine, Japan Institute of Sports Sciences, Kita-ku, Tokyo, Japan; University of Zurich, Switzerland

## Abstract

Muscle hardness is a mechanical property that represents transverse muscle stiffness. A quantitative method that uses ultrasound elastography for quantifying absolute human muscle hardness has been previously devised; however, its reliability and validity have not been completely verified. This study aimed to verify the reliability and validity of this quantitative method. The Young’s moduli of seven tissue-mimicking materials (*in vitro*; Young’s modulus range, 20–80 kPa; increments of 10 kPa) and the human medial gastrocnemius muscle (*in vivo*) were quantified using ultrasound elastography. On the basis of the strain/Young’s modulus ratio of two reference materials, one hard and one soft (Young’s moduli of 7 and 30 kPa, respectively), the Young’s moduli of the tissue-mimicking materials and medial gastrocnemius muscle were calculated. The intra- and inter-investigator reliability of the method was confirmed on the basis of acceptably low coefficient of variations (≤6.9%) and substantially high intraclass correlation coefficients (≥0.77) obtained from all measurements. The correlation coefficient between the Young’s moduli of the tissue-mimicking materials obtained using a mechanical method and ultrasound elastography was 0.996, which was equivalent to values previously obtained using magnetic resonance elastography. The Young’s moduli of the medial gastrocnemius muscle obtained using ultrasound elastography were within the range of values previously obtained using magnetic resonance elastography. The reliability and validity of the quantitative method for measuring absolute muscle hardness using ultrasound elastography were thus verified.

## Introduction

**Figure 1:**
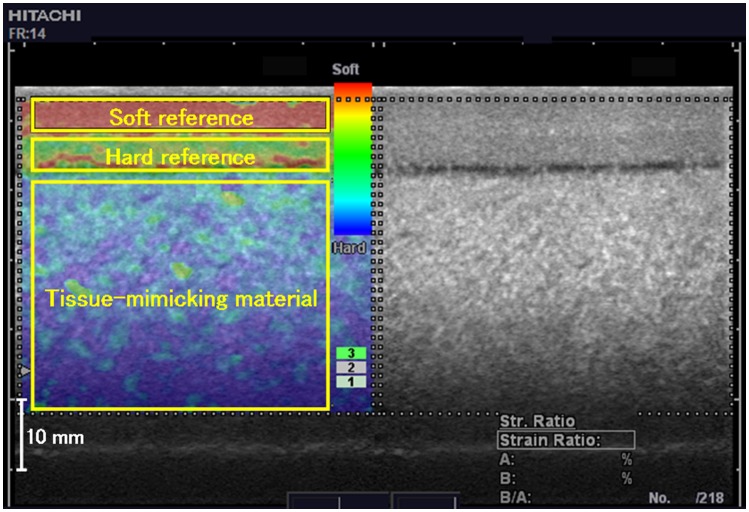
Typical ultrasound elastogram of the tissue-mimicking material. The image on the left is a color-coded elastogram superimposed on a conventional B-mode ultrasound image, whereas that on the right side is the B-mode ultrasound image. On the elastogram, a color-coded scale from red (soft) to blue (hard) and a numeric scale of 1–7 indicating the velocity of the compression–relaxation cycles are presented. Regions of interest within the soft mimicking reference (7 kPa), hard mimicking reference (30 kPa), and tissue-mimicking materials (50 kPa) are indicated by the yellow rectangles.

Muscle hardness is a mechanical property that represents transverse muscle stiffness, and it is different from muscle–tendon complex stiffness along the longitudinal axis of the muscle [1]. Ultrasound elastography, an imaging method for measuring tissue elasticity using a conventional ultrasound machine with special software [2], [3], has been used for measuring muscle hardness [4]–[8]. This method permits real-time measurements of the tissue strain induced by external freehand compression to the tissue using an ultrasound probe. The strain induced within a region of interest (ROI) in each tissue element is compared with the mean strain of all ROIs. Strain differences between each element are color-coded according to decreasing tissue strain, i.e., increasing tissue hardness, in the ascending order of red, yellow, green, and blue (Fig. 1). Furthermore, the strain ratio between any two regions among all ROIs can be calculated using the built-in software of the ultrasound system. The strain ratio between muscle and subcutaneous fat [4] or a reference material [6], [7], i.e., relative muscle hardness, has been measured in previous studies. Moreover, a new method for measuring absolute muscle hardness using ultrasound elastography was devised in a previous study [8]. The intra-investigator reliability of this method was verified in that study, but the inter-investigator reliability and validity remain unconfirmed. The purpose of this study was to confirm the reliability and validity of this method. Absolute muscle hardness was evaluated using Young’s modulus, one of the most relevant parameters for quantifying tissue elasticity [9].

## Methods

### Experiment 1

Commercially produced tissue-mimicking materials (width: 130 mm, depth: 80 mm, height: 40 mm) were prepared from agar, talc, ticking agent, and low-molecular-weight gel (OST Co., Ltd., Kashiwa-shi, Chiba, Japan). The Young’s moduli of these materials ranged from 20 kPa to 80 kPa (increments of 10 kPa), and these values were confirmed by the sales company using the mechanical displacement–load compression method with two types of devices (Rheo Meter CR-200D, Sun Scientific Corporation, Japan; Instron 3342, Instron, USA). To measure Young’s modulus using ultrasound elastography, two types of reference material (7 kPa: soft reference, 30 kPa: hard reference) were made from the same materials used for making the tissue-mimicking materials. The hard reference (width: 110 mm, depth: 60 mm, height: 5 mm) was placed on top of a tissue-mimicking material, and the soft reference (width: 110 mm, depth: 60 mm, height: 10 mm) was placed on the hard reference (Fig. 1). One investigator measured the Young’s moduli of the tissue-mimicking materials using ultrasound elastography (EUB-7500; Hitachi Medical Corporation, Chiyoda-ku, Tokyo, Japan) with a linear probe possessing a scanning frequency of 7.5 MHz. Slightly repetitive compression–relaxation cycles were applied to the materials with the handheld probe. The cyclic velocity was monitored in real time by a numeric scale indicator. The indicator classifies the cyclic velocity into a numeric scale of 1–7; the scale of 3–4 can be regarded as proper cyclic velocity [6], [8], [10]. For each measurement, 218 elastograms were stored at 14 Hz as cine loops in the memory of the ultrasound system, and the investigator selected the best elastogram for further analysis. The strains of the soft reference, hard reference, and tissue-mimicking materials were evaluated using the built-in software of the ultrasound system. The Young’s moduli of the tissue-mimicking materials were calculated by substituting its strain to the linear regression between the strains and the Young’s moduli of the two references. The investigator repeated the Young’s modulus measurements eight times for each type of tissue-mimicking material.

To confirm the intra-investigator reliability of the method, the coefficient of variation (CV) [11] and intraclass correlation coefficient (ICC) [1,1] [12] were calculated. To verify the validity of the method, Pearson’s correlation coefficient analysis and simple regression analysis were applied to determine the relationship between Young’s moduli obtained using the mechanical displacement–load compression method (*E*_Comp_) and ultrasound elastography (*E*_US_). In addition, the absolute percentage error between the Young’s moduli measured using the two methods was calculated as follows: absolute percentage error = [(*E*_US_ − *E*_Comp_)/*E*_Comp_]×100 [13].

### Experiment 2

Ten healthy males [age: 25.3±4.3 years, height: 172.5±5.7 cm, weight: 65.7±9.1 kg, mean ± standard deviation (SD)] voluntarily participated in this study, which was approved by the ethics review board of the Japan Institute of Sports Sciences (Permit Number: 013). Before the experiment, all subjects were fully informed of the experimental protocol as well as the purpose of the study. They provided written informed consent to participate in the experiment.

**Figure 2:**
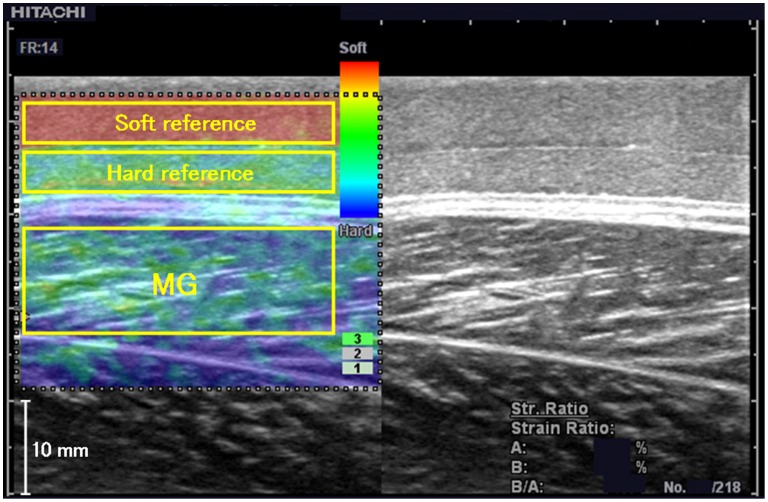
Typical ultrasound elastogram of the *in vivo* human medial gastrocnemius muscle (MG). The yellow rectangles are the regions of interest for the soft reference (7 kPa), hard reference (30 kPa), and MG.

The subjects lay prone on a bed and relaxed their lower limb muscles. The right foot of each subject was firmly secured to a custom-made duralumin footplate using four nonelastic straps. Three ankle joint positions were used: 20° dorsiflexion, anatomical position (angle between the tibia and sole: 90°), and 30° plantar flexion. A linear probe was positioned at the 30% proximal level of the distance between the popliteal crease and the center of the lateral malleolus in the proximodistal direction [14] and at the level where the muscle thickness of the medial gastrocnemius muscle (MG) was greatest in the mediolateral direction [15]. According to the procedures described in Experiment 1, the Young’s modulus of MG was measured using ultrasound elastography (Fig. 2). In brief, the Young’s modulus of MG was calculated by substituting its strain with the linear regression between the strains and the Young’s moduli of the two references. For each ankle joint position, two investigators alternately measured the muscle hardness of MG twice.

Intra- and inter-investigator reliability were evaluated using CV and ICC [1], [2] and CV and ICC [2,2], respectively. When calculating CV and ICC [2,2] for inter-investigator reliability, the mean value of two measurements by each investigator was used as the representative value.

The average of four measurements by the two investigators was taken as the Young’s modulus of MG in each ankle joint position. One-way repeated measures analysis of variance (ANOVA) was used to compare Young’s moduli between the ankle joint positions. If the results of ANOVA were significant, post hoc comparisons were performed using Bonferroni’s test.

The level of statistical significance was set at *P*<0.05. Unless otherwise specified, all data are presented as mean ± SD. All statistical analyses were performed using SPSS for Windows Version 11.0.1J (SPSS Japan, Tokyo, Japan).

## Results

### Experiment 1

**Figure 3:**
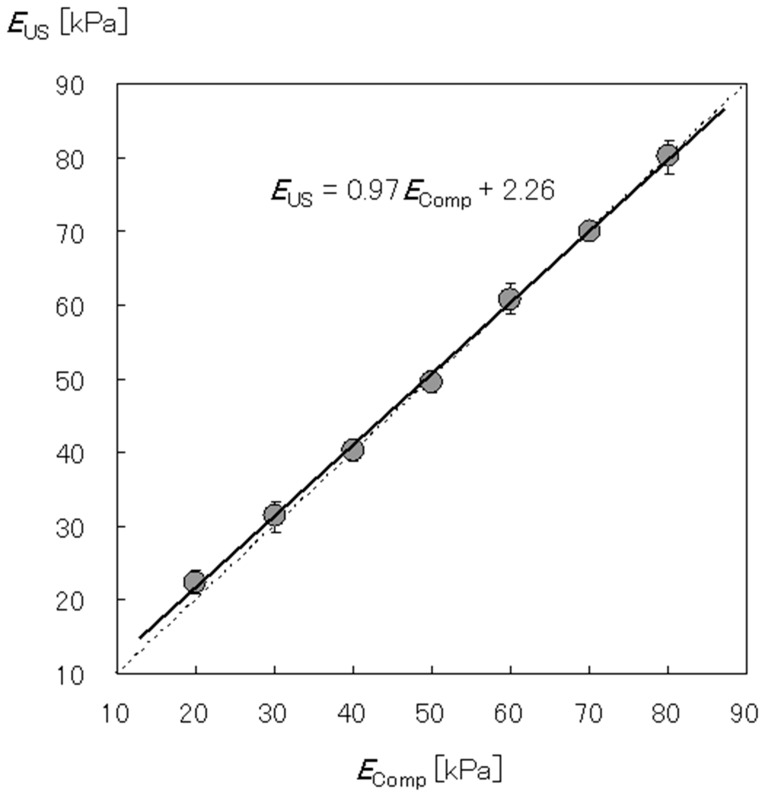
Comparison of the Young’s moduli of seven types of tissue-mimicking materials obtained using the mechanical displacement–load compression method (*E*_Comp_) and ultrasound elastography (*E*_US_). The two independent methods of calculating Young’s modulus were significantly correlated (r = 0.996, *P*<0.001).

CVs for the 20-, 30-, 40-, 50-, 60-, 70, and 80-kPa tissue-mimicking materials were 6.9%, 6.6%, 3.5%, 2.9%, 3.3%, 1.7%, and 2.9%, respectively. ICC [1,1] was 0.99 [95% confidence interval (CI), 0.98–1.00). A significant correlation was observed between *E*_Comp_ and *E*_US_ (r = 0.996, *P*<0.001), and a significant regression line (*E*_US_ = 0.97*E*_Comp_+2.26, *P*<0.001) was obtained using simple regression analysis (Fig. 3). The average absolute percentage errors for the 20-, 30-, 40-, 50-, 60-, 70, and 80-kPa tissue-mimicking materials were 12.1±7.2%, 7.0±4.1%, 2.5±2.4%, 2.0±2.1%, 3.0±1.7%, 1.4±0.9%, and 2.0±1.9%, respectively.

### Experiment 2

**Figure 4:**
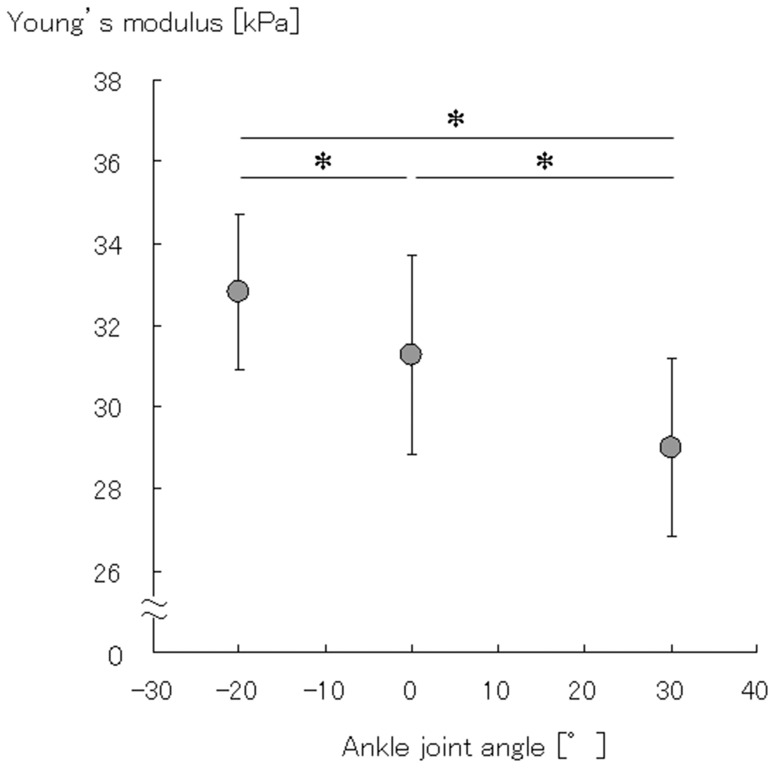
The Young’s modulus of the medial gastrocnemius muscle (MG). The Young’s modulus of MG was measured at 20° dorsiflexion, anatomical position, and 30° plantar flexion. The anatomical position of the ankle is considered as 0° (i.e., 90° between the tibia and the sole). Dorsiflexion is considered negative while plantar flexion is considered positive. Young’s modulus significantly decreased with plantar flexion (*P<0.05).

CVs for the two investigators were 3.6±2.3% and 5.2±2.9%, respectively, whereas ICCs [1], [2] were 0.89 (95% CI, 0.78–0.95) and 0.77 (95% CI, 0.52–0.89), respectively. CV between the two investigators was 3.1±2.4%, whereas ICC [2,2] was 0.89 (95% CI, 0.78–0.95). The Young’s modulus of MG significantly decreased with plantar flexion (*P*<0.05) (Fig. 4).

## Discussion

### Experiment 1

The maximum CV for the seven types of tissue-mimicking materials was 6.9%. On the basis of the criterion that CV less than 12% is regarded as acceptably low for a biological measurement [16], CV of this method can be regarded as acceptable. As a general rule, ICC over 0.75 is considered good [17], [18]. Therefore, ICC [1,1] for this experiment, which was calculated as 0.99, can be regarded as good. According to the CV and ICC [1,1] values, the intra-investigator reliability of this method for measuring absolute muscle hardness using ultrasound elastography was confirmed.

The correlation coefficient between *E*_Comp_ and *E*_US_ was 0.996, and the regression coefficient of the regression line was 0.97 (Fig. 3). Muthupillai et al. (1995) [19] measured the shear moduli of 10 different tissue-mimicking materials using the mechanical displacement–load compression method and magnetic resonance elastography (MRE). The correlation coefficient between the shear moduli derived using the two methods was 0.962, and the regression coefficient was 1.88. Suga et al. (2001) [20] also measured the shear moduli of five different tissue-mimicking materials using these same two methods and reported that the shear moduli obtained using the methods were similar (correlation coefficient = 0.993, regression coefficient = 1.05). In addition, Muthupillai et al. (1996) [21] measured the displacement of seven tissue-mimicking materials using an optical method and MRE and reported an excellent correlation coefficient between the two methods (r = 0.985). These results suggested that the correlation coefficient and regression coefficient obtained in the present study were similar to those reported previously. The absolute percentage error obtained in this study ranged from 1.4% to 12.1%. Suga et al. (2003) [22] measured the shear modulus of a tissue-mimicking material using two different MRE algorithms. The absolute percentage error for the two algorithms was determined to be 1.4% and 18.3%, respectively. This result indicated that the absolute percentage error obtained in this study was within the range of previously reported values. From the values of the correlation coefficient, regression coefficient, and absolute percentage error, it was considered that ultrasound elastography could measure absolute muscle hardness with a validity equivalent to that of MRE.

### Experiment 2

The CV values representing the intra-investigator (3.6% and 5.2%) and inter-investigator reliability (3.1%) were less than 12%, suggesting that these CV values were acceptably low for a biological measurement [16]. The ICC [1], [2] values for intra-investigator reliability were 0.77 and 0.89, which can be considered good [17], [18]. In addition, the ICC [2,2] value for inter-investigator reliability that was calculated as 0.89 can also be considered good.

The average Young’s modulus of MG obtained in the present study was approximately 30 kPa, and it significantly decreased with plantar flexion (Fig. 4). The shear moduli of MG measured using MRE were previously reported as 5.1 [22], 9.9 [23], and 24.9 kPa [24]. Assuming that Young’s modulus and shear modulus are related by a simple scale factor of 3: (Young’s modulus = 3 × shear modulus) [25], the Young’s moduli of MG in these studies could thus be calculated as 15.3, 29.7, and 74.7 kPa, respectively. If these values are accurate, then the Young’s moduli of MG calculated in this study were within the range obtained using MRE in previous studies. In addition, the decrease in the Young’s modulus of MG with plantar flexion was similar to that in previous studies that reported a decrease in the muscle hardness of the lateral gastrocnemius, tibialis anterior [26], and elbow flexor [27] muscles with passive muscle shortening.

### Conclusion

The present study investigated the reliability and validity of a method for quantifying absolute muscle hardness with ultrasound elastography, as described previously [8]. According to the results obtained from both tissue-simulated measurements (Experiment 1) and *in vivo* measurements (Experiment 2), the reliability and validity of this method were confirmed. It is expected that differences in muscle hardness between males and females, between individuals performing different athletic events, and between individuals with muscle conditions changed by fatigue can be quantified by the present method. Moreover, it will become possible to apply this method to the medical examination of regional damage such as muscle damage or muscle strain within an entire muscle.
